# Pharmacokinetics of the New Hepatitis C Virus NS3 Protease Inhibitor Narlaprevir following Single-Dose Use with or without Ritonavir in Patients with Liver Cirrhosis

**DOI:** 10.1128/AAC.01044-16

**Published:** 2016-11-21

**Authors:** V. Isakov, D. Koloda, N. Tikhonova, T. Kikalishvili, E. Krasavina, K. Lekishvili, I. Malaya, M. Ryska, M. Samsonov, V. Tolkacheva

**Affiliations:** a Department of Gastroenterology and Hepatology, Institute of Nutrition, Moscow, Russian Federation; b Medical Department, R-Pharm, Moscow, Russian Federation; c Academician G. Chapidze Emergency Cardiology Center, Tbilisi, Georgia; d Cardiological Clinic GULI, Tbilisi, Georgia; e Ascent Clinical Research Solutions, Moscow, Russian Federation; f Quinta Analytica, Prague, Czech Republic; g Federal Governmental Budget Healthcare Institution, Russian Academy of Science, Troitsk, Russian Federation

## Abstract

In this study we sought to evaluate narlaprevir (NVR) pharmacokinetics (PK) after a single dose with or without ritonavir (RTV) in cirrhotic versus healthy subjects. NVR at 200 mg was administered to 8 healthy and 8 cirrhotic subjects, and NVR at 100 mg with RTV at 100 mg was administered to 8 healthy and 8 cirrhotic subjects. PK analysis was performed. The geometric mean maximum concentration of a drug in serum (*C*_max_) and the area under the concentration-time curve from 0 to infinity (AUC_0–∞_) were 563.1 ng/ml and 4,701.8 ng · h/ml in cirrhotic patients versus 364.8 ng/ml and 1,917.1 ng · h/ml in healthy volunteers, respectively. The geometric mean ratios of the PK parameters of cirrhotic subjects to healthy volunteers were 1.54-fold (90% confidence interval [CI] = 1.05 to 2.27) for *C*_max_ and 2.45-fold (90% CI = 1.56 to 3.85) for AUC_0–∞_. The geometric mean *C*_max_ and AUC_0–∞_ in cirrhotic and healthy subjects were similar: 1,225.7 ng/ml for *C*_max_ and 15,213.1 ng · h/ml for AUC_0–∞_ in cirrhotic subjects and 1,178.9 ng/ml for *C*_max_ and 14,257.2 ng · h/ml for AUC_0–∞_ in healthy volunteers. The corresponding geometric mean ratios were 1.04 (90% CI = 0.67 to 1.62) for *C*_max_ and 1.07 (90% CI = 0.72 to 1.58) for AUC_0–∞_. Higher exposures in cirrhotic subjects were safe and well tolerated. We found that NVR exposures after a 200-mg single dose were higher in cirrhotic subjects than in healthy subjects and that a 100-mg single dose of NVR boosted with RTV at 100 mg resulted in no significant PK differences between cirrhotic and healthy subjects.

## INTRODUCTION

Narlaprevir (NVR) is a potent inhibitor of hepatitis C virus (HCV) NS3 protease with a *K_i_* of 7 ± 1 nM and a 90% inhibitory concentration (IC_90_) of ∼28 ng/ml for HCV genotype 1 replicon *in vitro* (1). Metabolic boosting with ritonavir (RTV) and administration under a fed condition allows favorable once-daily dosing of NVR (2). Phase II clinical trials have shown that the addition of 200 mg of NVR with RTV at 100 mg for 12 weeks to peginterferon and ribavirin significantly increases the rates of sustained virological response (SVR) up to 85% in treatment-naive noncirrhotic patients with chronic HCV genotype 1 infection (3). A phase III multicenter randomized placebo-controlled efficacy and safety study in 420 naive and treatment-experienced noncirrhotic patients has been recently completed in Russia.

HCV-related morbidity and mortality rates are increasing both globally and in Eastern Europe (4, 5) due to a substantial number of patients with advanced liver disease and liver cirrhosis. These patients are considered to be treated with priority since the eradication of HCV in them is associated with increased survival, interruption of the progression of the disease, and reversal of liver fibrosis (6). In advanced liver fibrosis and cirrhosis, liver function impairment results in disruption of metabolic pathways of many drugs, including direct-acting antivirals (DAAs) used for the treatment of HCV infection; therefore, compromised metabolism may both decrease antiviral activity and alter safety profiles of DAAs in these patients.

The objective of this study was to evaluate pharmacokinetics (PK) after a single oral dose of NVR alone and in combination with RTV in patients with compensated liver cirrhosis and in matched healthy controls.

(Some of the results of this study were presented at the Liver Meeting AASLD, 13 to 17 November 2015, San Francisco, CA, USA.)

## MATERIALS AND METHODS

This was an international two-part, open-label, parallel-group, single-dose phase I pharmacokinetic study conducted in two sites in Georgia and 1 site in Russia. A total of 32 adult subjects (6 women and 26 men) aged 18 to 75 years were included in the study: 16 patients with compensated cirrhosis Child-Pugh class A without active HCV infection and 16 healthy subjects, all Caucasians (Table 1). Subjects with cirrhosis were in stable condition, required to have documented history of hepatic disease other than chronic hepatitis C (CHC) diagnosed by liver biopsy, imaging techniques, and/or medical history of chronic liver disease, and had Child-Pugh scores of 5 or 6, consistent with Child-Pugh class A category. Healthy subjects, individually matched to cirrhotic patients based on gender, age, body mass index (BMI), and smoking status, were deemed healthy based on medical history, physical examination, laboratory tests, and 12-lead electrocardiograms and had negative test results for hepatitis B virus surface antigen and HCV antibodies. Key exclusion criteria for all subjects included positive screening tests results for hepatitis B and C viruses and human immunodeficiency virus, a history of drug sensitivity or drug abuse, prior use of medications contraindicated with ritonavir within 1 month prior to study drug administration, the presence of clinically significant comorbidities (other than liver failure for patients with cirrhosis), pregnancy, lactation, or pregnancy planning. The study was conducted in accordance with the principles of Good Clinical Practice, the Declaration of Helsinki, and local ethical and legal requirements. All participants provided informed consent forms approved by Independent Ethics Committees prior to the initiation of any screening or study-specific procedures.

**TABLE 1:** Demographic characteristics

Parameter	Part 1		Part 2		All participants (*n* = 32)
	Cirrhotic patients (*n* = 8)	Healthy subjects (*n* = 8)	Cirrhotic patients (*n* = 8)	Healthy subjects (*n* = 8)	
No. male (%)	6 (75)	6 (75)	7 (87)	7 (87)	26 (81)
No. female (%)	2 (25)	2 (25)	1 (13)	1 (13)	6 (19)
Mean age in yrs (SD)	56.4 (6.99)	53.9 (7.45)	49.8 (13.48)	49.0 (15.59)	52.3 (11.35)
Mean BMI (SD)	28.5 (4.18)	27.35 (4.40)	27.3 (4.65)	26.9 (4.14)	27.5 (4.18)

In part 1 of the study, 8 patients with compensated cirrhosis and 8 matched healthy adult subjects received single doses of NVR at 200 mg with 240 ml of water after a standard breakfast. The 200-mg NVR dose was chosen since this is the intended therapeutic dose. This dose is the approved marketed dose of NVR for the treatment of CHC genotype 1 in the Russian Federation.

**FIG 1:**
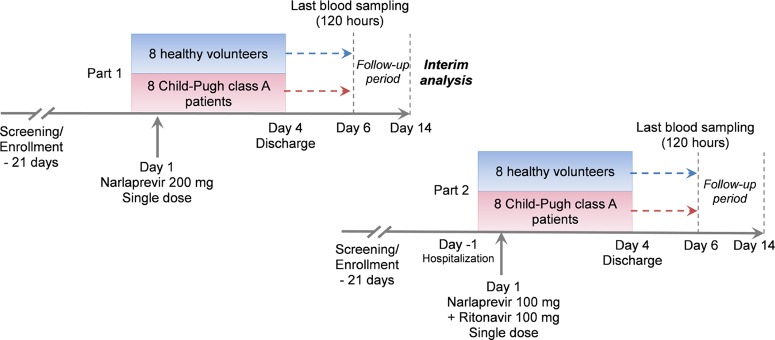
Study design.

In part 2 of the study, 8 patients with compensated cirrhosis and 8 healthy subjects received NVR at 100 mg in combination with RTV at 100 mg with 240 ml of water after a standard breakfast. The study design is depicted in Fig. 1. As an additional safety precaution, dosing in part 2 of the study was conducted after an interim analysis of the part 1 results.

Intensive blood sampling for NVR and RTV (for part 2) concentrations was performed 0.25, 0.5, 0.75, 1, 1.5, 2, 2.5, 3, 4, 8, 12, 24, 36, 48, 72, 96, and 120 h after dosing. Blood samples were collected into EDTA tubes and stored on ice until centrifugation, and plasma samples were stored at −20°C until analysis. Narlaprevir and ritonavir levels in human matrix were determined through a validated high-performance method in a certified analytical laboratory, Quinta-Analytica, under Good Laboratory Practices according to EU regulations. The method was based on tandem mass spectrometry (high-pressure liquid chromatography/electrospray ionization/tandem mass spectrometry) in positive mode performed after the removal of proteins in the sample by precipitation with methanol.

A noncompartmental model was used for PK analysis of NVR and RTV in plasma and urine (data are not shown). PK parameters were calculated using Phoenix WinNonlin software (version 6.3). The maximum observed plasma concentration (*C*_max_) and the time to *C*_max_ achievement (*T*_max_), the terminal half-life (*t*_1/2_), and the area under the concentration-time curve from time zero to infinity (AUC_0–∞_) were calculated for NVR; the *C*_max_, the area under the concentration-time curve from time zero to the last measurement (AUC_0–last_), and the *T*_max_ were calculated for RTV. Simulation of PK parameters for NVR at steady state was performed using the WinNonlin module “NonParametric Superposition.” The *C*_max_ for steady state (*C*_max ss_), the AUC_tau_ (tau = 24 h), the minimal predose concentration (*C*_trough_), and the accumulation index were calculated. The lower limit of quantification (LLOQ) for NVR in plasma was 10.0 ng/ml, with an interassay accuracy of 103.94% and an interassay precision matching a 7.51% coefficient of variation (CV) at LLOQ.

Safety and tolerability were evaluated based on adverse event monitoring, vital signs measurements, physical examinations, two-lead ECG assessments, and laboratory tests. All cirrhotic patients and healthy volunteers (32 in all) completed the study and were included in the analysis. The total duration of each patient's participation in the study was up to 35 days. Geometric means (GMEAN) were calculated for single-dose PK parameters and for steady-state simulation results, both with 90% CIs. The narlaprevir *C*_trough_ values in part 2 of the study were compared to the IC_90_ against HCV in order to demonstrate the achievement of therapeutic concentrations in plasma. The IC_90_ value was not protein adjusted.

## RESULTS AND DISCUSSION

**FIG 2:**
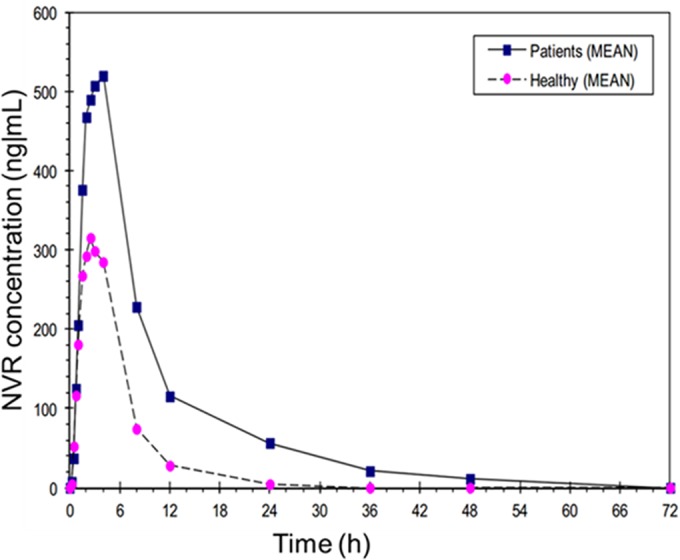
Concentration-time curves for cirrhotic patients and healthy volunteers after 200-mg single-dose narlaprevir treatment (part 1) on a normal scale.

**FIG 3:**
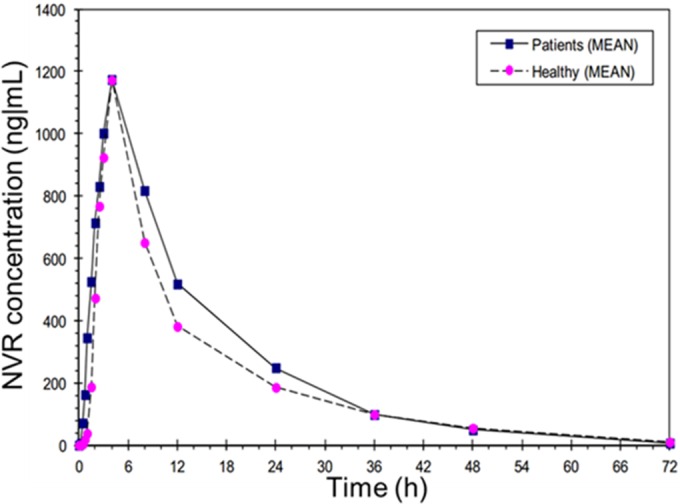
Concentration-time curves for cirrhotic patients and healthy volunteers after narlaprevir (100 mg) plus ritonavir 100-mg single dosing (part 2) on a normal scale.

Healthy subjects were individually matched to cirrhotic patients; therefore, subject demographics were well balanced between cohorts in terms of age, body weight, sex, smoking status, and race/ethnicity (all were Caucasians). The mean concentration-time curves after single dosing of NVR at 200 mg in part 1 of the study and NVR plus RTV in part 2 for patients with cirrhosis and healthy subjects are shown in Fig. 2 and 3 on a normal scale.

The GMEAN *C*_max_ and AUC_0–∞_ values were 1.54 and 2.45 times higher in patients with compensated cirrhosis versus healthy subjects, respectively, in part 1 of the study (Tables 2 and 3). The simulated steady-state (tau = 24 h) GMEAN *C*_max ss_ and AUC_tau_ values in patients with compensated cirrhosis were 1.67 and 2.37 times higher than in healthy subjects, respectively (Table 4). Taking into account the increase in NVR exposure in patients with cirrhosis compared to healthy volunteers in part 1 of the study and further expected increase due to RTV boosting, NVR dose was reduced to 100 mg in part 2 of the study.

**TABLE 2:** Plasma PK parameters after single-dose administration of narlaprevir (part 1) and narlaprevir with ritonavir (part 2) in patients with compensated cirrhosis and in healthy subjects^*a*^

Parameter	Part 1 (NVR 200 mg)		Part 2 (NVR 100 mg/RTV 100 mg)	
	Cirrhotic patients (*n* = 8)	Healthy subjects (*n* = 8)	Cirrhotic patients (*n* = 8)	Healthy subjects (*n* = 8)
*t*_1/2_ (h)	9.3 (44.2)	2.6 (53.6)	10.4 (36.6)	12.2 (24.8)
*T*_max_ (h)	4.0 (48.7)	1.5 (49.2)	4.0 (52.0)	4.0 (39.8)

a *t*_1/2_ and *T*_max_ values are both expressed as medians, with the coefficients of variation indicated in parentheses. NVR, narlaprevir; RTV, ritonavir.

**TABLE 3:** Plasma PK parameters after single-dose administration of narlaprevir (part 1) and narlaprevir with ritonavir (part 2) in patients with compensated cirrhosis and in healthy subjects^*a*^

Investigated regimen	Parameter	GMEAN		P/H	90% CI	
		Cirrhotic patients (*n* = 8)	Healthy subjects (*n* = 8)		Lower	Upper
NVR at 200 mg (part 1)	AUC_0–∞_ (ng · h/ml)	4,701.8	1,917.1	2.45	1.56	3.85
	*C*_max_ (ng/ml)	563.1	364.8	1.54	1.05	2.27
NVR at 100 mg + RTV at 100 mg (part 2)	AUC_0–∞_ (ng · h/ml)	15,213.1	14,257.2	1.07	0.72	1.58
	*C*_max_ (ng/ml)	1,225.7	1,178.9	1.04	0.67	1.63

a *C*_max_ and AUC_0–∞_ are presented as arithmetic means. P/H, patient/healthy subject ratio; NVR, narlaprevir; RTV, ritonavir.

**TABLE 4:** Simulated steady-state PK after single-dose administration of narlaprevir (part 1) and narlaprevir with ritonavir (part 2) in patients with compensated cirrhosis and in healthy subjects^*a*^

Investigated regimen	Parameter	GMEAN		P/H	90% CI	
		Cirrhotic patients (*n* = 8)	Healthy subjects (*n* = 8)		Lower	Upper
NVR at 200 mg (part 1)	AUC_tau_ (ng · h/ml)	4,607.2	1,944.0	2.37	1.50	3.74
	*C*_max ss_ (ng/ml)	618.9	370.5	1.67	1.14	2.45
NVR at 100 mg + RTV at 100 mg (part 2)	AUC_tau_ (ng · h/ml)	15,105.7	14,115.9	1.07	0.72	1.58
	*C*_max ss_ (ng/ml)	1,450.6	1,378.1	1.05	0.68	1.62

a AUC_tau_, area under narlaprevir concentration-time curve at steady state; *C*_max ss_, maximum narlaprevir concentration at steady state; P/H, patient/healthy subject ratio; GMEAN, geometric least square mean; NVR, narlaprevir; RTV, ritonavir.

The reduced single 100-mg NVR dose in combination with RTV at 100 mg in part 2 of the study led to significantly higher NVR concentrations than those observed in part 1, both in healthy volunteers and in patients with compensated cirrhosis. There were no significant differences in plasma NVR exposures between patients with compensated cirrhosis and healthy subjects after single-dose NVR at 100 mg in combination with RTV at 100 mg; the GMEAN *C*_max_ and AUC_0–∞_ values were 1.04 and 1.07 in patients with compensated cirrhosis versus healthy subjects, respectively. In steady-state simulations, the GMEAN *C*_max ss_ and AUC_tau_ values were 1.05 and 1.07, respectively, in patients with compensated cirrhosis versus healthy subjects (Table 4).

**FIG 4:**
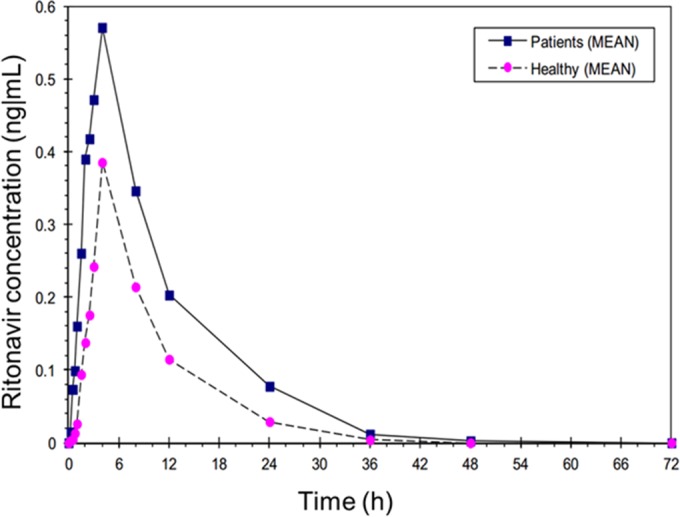
Mean ritonavir concentrations after single-dose administration (narlaprevir at 100 mg plus ritonavir at 100 mg) in patients with compensated cirrhosis and healthy volunteers in part 2 of the study.

The ritonavir PK parameters were determined for cirrhotic patients and healthy volunteers in part 2 of the study. The concentration-time curves demonstrated similar patterns for cirrhotic patients and healthy volunteers; however, the achieved maximum concentration and the AUC were 44.6 and 63.6% higher, respectively, in the compensated cirrhosis group (Fig. 4). All ritonavir PK parameters demonstrated high variability (Table 5).

**TABLE 5:** Plasma PK parameters of ritonavir after single-dose administration of narlaprevir with ritonavir (part 2) in patients with compensated cirrhosis and in healthy subjects^*a*^

Parameter	Cirrhotic patients (*n* = 8)		Healthy subjects (*n* = 8)		P/H
	GMEAN	CV (%)	GMEAN	CV (%)	
*C*_max_ (ng/ml)	0.556	80.5	0.384	20.3	1.45
AUC_0–last_ (ng · h/ml)	5.198	66.1	3.178	21.7	1.64
*T*_max_ (h)	4.0	52.1	4.2	34.4	0.9

a *C*_max_ and AUC values are presented as arithmetic means, and *T*_max_ values are presented as medians. P/H, patient/healthy subject ratio; CV, coefficient of variation.

**FIG 5:**
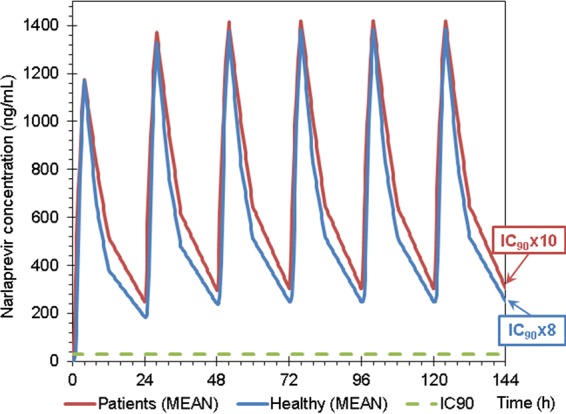
Mean narlaprevir concentrations at steady state in patients with compensated cirrhosis and in healthy volunteers in part 2 of the study (narlaprevir at 100 mg plus ritonavir at 100 mg) compared to the IC_90_ level (28 ng/ml).

**FIG 6:**
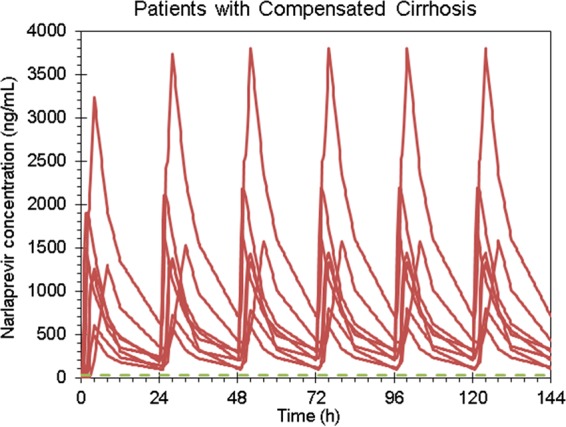
Individual narlaprevir concentrations at steady state in patients with compensated cirrhosis in part 2 of the study (narlaprevir at 100 mg plus ritonavir at 100 mg) compared to the IC_90_ level (28 ng/ml).

**FIG 7:**
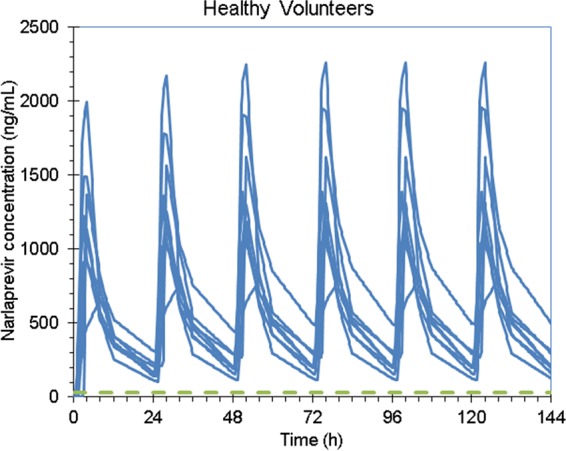
Individual narlaprevir concentrations at steady state in healthy volunteers in part 2 of the study (narlaprevir at 100 mg plus ritonavir at 100 mg) compared to the IC_90_ level (28 ng/ml).

Narlaprevir *C*_trough_ plasma values at steady state (simulation for NVR at 100 mg with RTV at 100 mg using once-daily dosing) were at least 8 times higher than the IC_90_ both in patients with compensated cirrhosis and in healthy volunteers (Fig. 5, 6, and 7). Therefore, the 100-mg dose of NVR boosted by RTV at 100 mg can theoretically be sufficient for the treatment of HCV-infected patients with compensated cirrhosis, although this concept requires further clinical investigation.

NVR accumulation at steady-state was low in patients with compensated cirrhosis in both parts of the study. The accumulation index was 1.23 (CV = 18.7%) in part 1 and 1.21 (CV = 17.7%) in part 2 of the study.

Cirrhotic patients and healthy volunteers tolerated NVR well both in monotherapy and in combination with RTV. Of 32 subjects, 2 reported four adverse events in all (Table 6). No clinically significant treatment-emergent laboratory abnormalities were observed. There were no clinically significant changes from baseline in vital signs.

**TABLE 6:** Adverse events

Adverse event^*a*^	% (*n*)	
	Cirrhotic patients (*n* = 16)	Healthy volunteers (*n* = 16)
Supraventricular tachycardia	6.3 (1)	0
Atrial fibrillation paroxysm	6.3 (1)	0
Premature ventricular contractions	6.3 (1)	0
Mild somnolence	6.3 (1)	0

a These first adverse events occurred in the same patient on day 4 after a single dose of narlaprevir at 200 mg (part 1), including premature supraventricular contractions, which was considered serious and possibly related to study treatment. It developed in a 67-year-old patient with arterial hypertension and previous episodes of premature ventricular contractions in her medical history. All adverse events in this patient resolved with appropriate medical treatment.

The study was designed to assess the impact of mild hepatic impairment on NVR pharmacokinetics after single dosing alone and in combination with RTV. Narlaprevir plasma exposures in patients with cirrhosis (Child-Pugh class A) were found to be significantly higher than in healthy volunteers (*C*_max_ values were 1.54 times higher, and AUC values were 2.45 times higher) after single-dose administration. Coadministration of NVR in a reduced dose with RTV in the second part of the study resulted in higher NVR exposure values compared to those after single NVR dosing in part 1 of the study, both in patients with mild hepatic impairment and in healthy volunteers. There were no differences in plasma NVR exposure between patients with cirrhosis and healthy volunteers (*C*_max_ values were 1.04 times higher, and AUC values were 1.07 times higher).

It is clear that chronic liver disease can significantly affect hepatic drug metabolism and elimination, thereby potentially affecting the pharmacokinetics of drugs that are primarily eliminated by the liver. Another explanation is that RTV can affect NVR metabolism and elimination. Narlaprevir metabolism occurs in the liver, and biliary excretion is the major route for elimination. NVR is a substrate for the efflux transporter P-gp and the cytochrome P450 enzyme CYP3A4; the enhancing effect of RTV may be attributed to the inhibition of both P-gp and CYP3A4. A phase I study (P05065) has shown that only 3.1% of the radioactivity was found in urine, whereas 81.1% of the radioactivity was detected in feces after single oral administration of radiolabeled NVR at 400 mg as a single agent (data not shown). It is noteworthy that the percentage of detected radioactivity in urine and feces changed when RTV was coadministered with NVR; radioactivity in the urine and feces accounted for 33.5 and 55.9% of the dose, respectively, after a single oral dose of radiolabeled NVR at 400 mg with RTV at 100 mg. These results are consistent with data from our study: NVR exposure in patients with cirrhosis and mild hepatic insufficiency was significantly higher than in healthy volunteers when NVR was administered as monotherapy. This can be explained by the fact that NVR given as a single agent is primarily eliminated via the liver and the gastrointestinal tract, whereas elimination via urine becomes more relevant after coadministration with RTV.

In order to confirm that the reduced dose of NVR coadministered with RTV will be sufficient to achieve feasible efficacy of NVR-containing regimens in patients with or without cirrhosis, we compared actual NVR exposure values with the IC_90_ against the HCV replicon *in vitro*. Steady-state narlaprevir *C*_trough_ plasma values (simulation for NVR at 100 mg with RTV at 100 mg once-daily dosing) were shown to be at least 8 times higher than the IC_90_ both in patients with compensated cirrhosis and in healthy volunteers (Fig. 5 to 7). However, despite these promising observations, there is an unmet need to conduct clinical studies in HCV-infected patients with cirrhosis in order to make definite recommendations regarding a feasible NVR dose in this category of patients.

Human liver microsomes from patients with cirrhosis or patients with both cirrhosis and cholestasis demonstrated approximately 25 to 90% lower expression of CYP3A4 activity (7). CYP3A4 protein concentrations and enzymatic activity were also lower in patients with noncholestatic liver cirrhosis (8, 9). Therefore, the prominent effect of mild hepatic impairment on the exposure to NVR in monotherapy can be due to decreased CYP3A4 activity, since this is the primary route of NVR metabolism. In the presence of RTV, the booster significantly inhibiting NVR metabolism both in patients with cirrhosis and in healthy subjects, differences in NVR exposure between these groups become less prominent.

Cirrhotic patients have been shown to have increased exposures of other HCV protease inhibitors. The mean steady-state AUC of simeprevir was 2.4-fold higher in HCV-uninfected subjects with moderate hepatic impairment (Child-Pugh class B) and 5.2-fold higher in HCV-uninfected subjects with severe hepatic impairment (Child-Pugh class C) compared to HCV-uninfected subjects with normal hepatic function (10). The mean AUCs of boceprevir active diastereomer (SCH534128) were 32 and 45% higher in subjects with moderate and severe hepatic impairment, respectively, than in subjects with normal hepatic function (11). Mild hepatic impairment had only a minimal effect on steady-state asunaprevir pharmacokinetics (21% decrease compared to controls), whereas moderate and severe hepatic impairment significantly increased asunaprevir plasma exposures (9.83- and 32.1-fold increase in AUC, respectively) (12). It is noteworthy that telaprevir steady-state exposures decreased by 15 and 46% in HCV-negative subjects with mild (Child-Pugh class A) and moderate (Child-Pugh class B) hepatic impairment, respectively, compared to healthy subjects (13).

Increased exposures of another ritonavir-boosted protease inhibitor, paritaprevir, were demonstrated in patients with moderate and severe hepatic impairment compared to healthy volunteers (14). That study evaluated the effect of various degrees of hepatic impairment on paritaprevir, ritonavir, ombitasvir, and dasabuvir pharmacokinetics after a single oral dose of this combination in patients with nonviral cirrhosis and in subjects with normal liver function. Paritaprevir AUC values decreased by 29% in patients with mild hepatic failure and increased by 62 and 945%, respectively, in subjects with moderate and severe hepatic impairment compared to subjects with normal hepatic function (14, 15). Grazoprevir exposures were increased 62% in patients with mild (Child-Pugh class A) and 388% in patients with moderate (Child-Pugh class B) hepatic impairment relative to those with no hepatic impairment (16). The difference in our study is that all components of the combination, including ritonavir, were given in a single therapeutic dose. Only NVR was given in the first part of our study, where a significant increase in NVR exposure was detected, and then a reduced dose was given in combination with ritonavir, whereas the therapeutic dose of NVR for noncirrhotic patients (200 mg) in combination with RTV was not investigated in cirrhotic subjects.

Subtype 1b is the most prevalent genotype of HCV in many countries across Central and Eastern Europe, Asia, and the Middle East (17), where interferon-based treatment regimens are still used due to cost and acceptable efficacy. Recently, narlaprevir has been approved by the Russian Ministry of Health for the treatment of naive and treatment-experienced patients with CHC genotype 1 in combination with RTV, peginterferon, and ribavirin; this combination demonstrated a very high SVR rate but was still associated with adverse events typical to interferon-based regimens. Therefore, in the near future, NVR may become a part of interferon-free all-oral regimens with other DAAs that will be available in some regional markets, including Russia and Turkey. The most promising combination among different DAA regimens could be NVR/RTV plus an NS5A inhibitor or an HCV polymerase inhibitor. The combination of NVR/RTV with other DAA classes could be very effective, particularly in genotype 1b patients.

Improved NVR exposure by RTV plays an important role in sustaining the antiviral efficacy of narlaprevir. So far, no adverse events were solely attributed to ritonavir in the NVR clinical program. The coadministration of RTV with NVR may also facilitate multidrug regimens suitable for patients with HCV and HIV coinfection, where ritonavir is commonly used as a pharmacologic booster. A limitation associated with ritonavir coadministration is the increased risk for drug-drug interactions (DDIs) at the level of the CYP3A4 enzyme. However, the DDI potential of ritonavir is also well characterized, and guidance for the coadministration of this medication is easily determined according to the label.

In conclusion, a single dose of NVR administered as a single agent and in combination with RTV was well tolerated both by patients with compensated cirrhosis and by healthy volunteers. Narlaprevir exposures after a single dose of 200 mg were higher in patients with compensated cirrhosis than in healthy subjects. No significant effect on NVR exposure in patients with compensated cirrhosis compared to healthy subjects was found when NVR at 100 mg was coadministered with RTV at 100 mg. The narlaprevir *C*_trough_ at steady state was at least 8 times higher than the corresponding IC_90_ values both in patients with compensated cirrhosis and in healthy volunteers, although additional studies in patients with HCV-related cirrhosis are essential in order to determine a safe and effective NVR dose for this population.

## References

[1] Tong X, Arasappan A, Bennett F, Chase R, Feld B, Guo Z, Hart A, Madison V, Malcolm B, Pichardo J, Prongay A, Ralston R, Skelton A, Xia E, Zhang R, Njoroge FG 2010 Preclinical characterization of the antiviral activity of SCH 900518 (narlaprevir), a novel mechanism-based inhibitor of hepatitis C virus NS3 protease. Antimicrob Agents Chemother 54:2365–2370. doi:10.1128/AAC.00135-10. 20308381 PMC2876368

[2] Reesink H, Bergmann J, de Bruijne J, Weegink C, van Lier J, van Vliet A, Keung A, Li J, O'Mara E, Treitel M, Hughes E, Janssen H, de Knegt R 2009 Safety and antiviral activity of SCH 900518 administered as monotherapy and in combination with peginterferon alfa-2b to naive and treatment-experienced HCV-1-infected patients. J Hepatol 50:S35–S36. doi:10.1016/S0168-8278(09)60088-X.

[3] Vierling JM, Poordad FF, Lawitz E, Ghalib RH, Lee WM, Ravendhran N, Galati GS, Bacon BR, Flamm SL, Balart LA, Freilich B, Schiff ER, Jacobson IM, Kwo PY, Gordon SC, Sulkowski MS, Jiang R, Boparai N, Chaudhri EI, Treitel MA, Hughes EA, Brass CA, Albrecht JK 2011 Once daily Narlaprevir (NVR; SCH 900518) and Ritonavir (RTV) in combination with peginterferon alfa-2b/ribavirin (PR) for 12 weeks plus 12 weeks PR in treatment-naive patients with HCV genotype 1 (G1): SVR results from NEXT-1, a phase 2 study. Hepatology 54:1437A.

[4] Saraswat V, Norris S, Knegt RJ, Sanchez Avila JF, Sonderup M, Zuckerman E, Arkkila P, Stedman C, Acharya S, Aho I, Anand AC, Andersson MI, Arendt V, Baatarkhuu O, Barclay K, Ben-Ari Z, Bergin C, Bessone F, Blach S, Blokhina N, Brunton CR, Choudhuri G, Chulanov V, Cisneros L, Croes EA, Dahgwahdorj YA, Dalgard O, Daruich JR, Dashdorj NR, Davaadorj D, de Vree M, Estes C, Flisiak R, Gadano AC, Gane E, Halota W, Hatzakis A, Henderson C, Hoffmann P, Hornell J, Houlihan D, Hrusovsky S, Jarcuska P, Kershenobich D, Kostrzewska K, Kristian P, Leshno M, Lurie Y, Mahomed A, Mamonova N, Mendez-Sanchez N, Mossong J, Nurmukhametova E, Nymadawa P, Oltman M, Oyunbileg J, Oyunsuren Ts, Papatheodoridis G, Pimenov N, Prabdial-Sing N, Prins M, Puri P, Radke S, Rakhmanova A, Razavi H, Razavi-Shearer K, Reesink HW, Ridruejo E, Safadi R, Sagalova O, Sanduijav R, Schreter I, Seguin-Devaux C, Shah SR, Shestakova I, Shevaldin A, Shibolet O, Sokolov S, Souliotis K, Spearman CW, Staub T, Strebkova EA, Struck D, Tomasiewicz K, Undram L, van der Meer AJ, van Santen D, Veldhuijzen I, Villamil FG, Willemse S, Zuure FR, Silva MO, Sypsa V, Gower E 2015 Historical epidemiology of hepatitis C virus (HCV) in select countries, volume 2. J Viral Hepat 22(Suppl 1):6–25. doi:10.1111/jvh.12350. 25560839

[5] Hope VD, Eramova I, Capurro D, Donoghoe MC 2013 Prevalence and estimation of hepatitis, band C infections in the WHO European Region: a review of data focusing on the countries outside the European Union and the European Free Trade Association. Epidemiol Infect 2:270–286. 10.1017/S0950268813000940 PMC3891474 23714072

[6] European Association for the Study of the Liver. 2015 EASL recommendations on treatment of hepatitis C. J Hepatol 63:199–236. doi:10.1016/j.jhep.2015.03.025. 36464532

[7] Johnson TN, Boussery K, Rowland-Yeo K, Tucker GT, Rostami-Hodjegan A 2010 A semi-mechanistic model to predict the effects of liver cirrhosis on drug clearance. Clin Pharmacokinet 49:189–206. doi:10.2165/11318160-000000000-00000. 20170207

[8] George J, Murray M, Byth K, Farrell GC 1995 Differential alterations of cytochrome P450 proteins in livers from patients with severe chronic liver disease. Hepatology 21:120–128. doi:10.1002/hep.1840210121. 7806144

[9] Lucas D, Berthou F, Dréano Y, Lozac'h P, Volant A, Ménez JF 1993 Comparison of levels of cytochromes P-450, CYP1A2, CYP2E1, and their related monooxygenase activities in human surgical liver samples. Alcohol Clin Exp Res 17:900–905. doi:10.1111/j.1530-0277.1993.tb00861.x. 8214432

[10] U.S. Food and Drug Administration. 2013 Olysio (simeprevir): FDA prescribing information. U.S. Food and Drug Administration, Washington, DC http://www.accessdata.fda.gov/drugsatfda_docs/label/2014/205123s003lbl.pdf.

[11] U.S. Food and Drug Administration. 2011 Victrelis (boceprevir): FDA prescribing information. U.S. Food and Drug Administration, Washington, DC http://www.accessdata.fda.gov/drugsatfda_docs/label/2011/202258lbl.pdf.

[12] Eley T, He B, Chang I, Colston E, Child M, Bedford W, Kandoussi H, Pasquinelli C, Marbury TC, Bertz RJ 2015 The effect of hepatic impairment on the pharmacokinetics of asunaprevir, an HCV NS3 protease inhibitor. Antivir Ther 20:29–37. 24704773 10.3851/IMP2773

[13] U.S. Food and Drug Administration. 2011 Incivek (telaprevir): FDA prescribing information. U.S. Food and Drug Administration, Washington, DC http://www.accessdata.fda.gov/drugsatfda_docs/label/2011/201917lbl.pdf.

[14] Khatri A, Menon RM, Marbury TC, Lawitz EJ, Podsadecki TJ, Mullally VM, Ding B, Awni WM, Bernstein BM, Dutta S 2015 Pharmacokinetics and safety of co-administered paritaprevir plus ritonavir, ombitasvir, and dasabuvir in hepatic impairment. J Hepatol 63:805–812. doi:10.1016/j.jhep.2015.05.029. 26070406

[15] U.S. Food and Drug Administration. 2014 Viekira Pak (ombitasvir, paritaprevir, ritonavir, and dasabuvir): FDA prescribing information. U.S. Food and Drug Administration, Washington, DC http://www.accessdata.fda.gov/drugsatfda_docs/label/2014/206619lbl.pdf.

[16] MacBrayne CE, Kiser JJ 2016 Pharmacologic considerations in the treatment of hepatitis C virus in persons with HIV. Clin Infect Dis 63(Suppl 1):12–23. doi:10.1093/cid/ciw220. PMC4928451 27363437

[17] Gower E, Estes C, Blach S, Razavi-Shearer K, Razavi H 2014 Global epidemiology and genotype distribution of the hepatitis C virus infection. J Hepatol 61:S45–S57. doi:10.1016/j.jhep.2014.07.027. 25086286

